# Nicotinamide Mononucleotide: Exploration of Diverse Therapeutic Applications of a Potential Molecule

**DOI:** 10.3390/biom9010034

**Published:** 2019-01-21

**Authors:** Saikat Kumar Poddar, Ali Ehsan Sifat, Sanjana Haque, Noor Ahmed Nahid, Sabiha Chowdhury, Imtias Mehedi

**Affiliations:** 1 Department of Clinical Pharmacy and Pharmacology, Faculty of Pharmacy, University of Dhaka, Dhaka 1000, Bangladesh; ali.ehsan.sifat@gmail.com (A.E.S.); haquesanjana@gmail.com (S.H.); noor.nahid@gmail.com (N.A.N.); sabihanabila@gmail.com (S.C.); 2 Department of Pharmaceutical Chemistry, Faculty of Pharmacy, University of Dhaka, Dhaka 1000, Bangladesh; imtiasmehedi@gmail.com

**Keywords:** ageing, Alzheimer’s disease, diabetes, ischemic preconditioning, nicotinamide mononucleotide, obesity

## Abstract

Nicotinamide mononucleotide (NMN) is a nucleotide that is most recognized for its role as an intermediate of nicotinamide adenine dinucleotide (NAD+) biosynthesis. Although the biosynthetic pathway of NMN varies between eukaryote and prokaryote, two pathways are mainly followed in case of eukaryotic human—one is through the salvage pathway using nicotinamide while the other follows phosphorylation of nicotinamide riboside. Due to the unavailability of a suitable transporter, NMN enters inside the mammalian cell in the form of nicotinamide riboside followed by its subsequent conversion to NMN and NAD+. This particular molecule has demonstrated several beneficial pharmacological activities in preclinical studies, which suggest its potential therapeutic use. Mostly mediated by its involvement in NAD+ biosynthesis, the pharmacological activities of NMN include its role in cellular biochemical functions, cardioprotection, diabetes, Alzheimer’s disease, and complications associated with obesity. The recent groundbreaking discovery of anti-ageing activities of this chemical moiety has added a valuable essence in the research involving this molecule. This review focuses on the biosynthesis of NMN in mammalian and prokaryotic cells and mechanism of absorption along with the reported pharmacological activities in murine model.

## 1. Introduction

Nicotinamide mononucleotide (NMN) or Nicotinamide-1-ium-1-β-D-ribofuranoside 5′-phosphate is a type of bioactive nucleotide which is naturally formed by the reaction between a phosphate group and a nucleoside containing ribose and nicotinamide [1]. Generally, it exists in two anomeric forms namely alpha and beta. The beta anomer is the active form between these two with a molecular weight of 334.221 g/mol [2]. NMN is naturally abundant in various types of food [3]. Vegetables like broccoli, cabbage contain 0.25–1.12 and 0.0–0.90 mg NMN/100 gm, fruits like avocado, tomato contain 0.36–1.60 and 0.26–0.30 mg NMN/100 gm, whereas raw beef has 0.06–0.42 mg NMN/100 gm [3]. NMN is also used as a substrate for prokaryotic enzymes like NadM in *Methanobacterium thermoautotrophicum*, NadR in *Haemophilus influenza*, NadM/Nudix in *Francisella tularensis* [4].

In human cells, NMN is available as a source of cellular energy. Not long ago, this molecule was only known for its activity as an intermediate in nicotinamide adenine dinucleotide (NAD+) biosynthesis. During this biosynthetic process of NAD+, NMN acts as an important substrate for enzymes like nicotinamide mononucleotide adenylyltransferase 1 or NMNAT 1 of nuclear origin and NMNAT 3 of mitochondrial origin that helps in the enzymatic conversion to NAD+ in human [5]. Recently, preclinical studies have demonstrated diversified pharmacological activities of NMN in cardiac and cerebral ischemia, Alzheimer’s disease, diet- and age-induced type 2 diabetes, and obesity, all of which are linked up to the deficiency of NAD+ [6,7,8]. Camacho-Pereira et al. have shown that an increased level of NAD+ consuming enzymes e.g., NAD+ dependent acetylase (Sirtuins), poly ADP-ribose polymerase (PARP), NADase (CD38) contribute to the decline of NAD+ with age [9]. In mammalian cells, CD38, a type of cell surface NADase enzyme, causes breakdown of NAD+ to form nicotinamide and (cyclic-)ADP-ribose [10]. On the other hand, expenditure of NAD+ helps PARP to produce branched ADP-ribose polymers that help in DNA repairing [11]. Another group of NAD+ consuming enzymes, sirtuins (SIRT 1-7) performs different functions by consuming NAD+. Apart from deacetylation, which is the most common NAD+ mediated function of sirtuins, other functions like desuccinylase, demalonylase, lipoamidase, and deglutarylase enzymatic activity are noteworthy that help in the cellular adaptation of energy deficit and improvement of metabolic function [12]. Administration of NMN can compensate for the deficiency of NAD+ caused by these NAD+ consuming enzymes.

NMN shares similar properties like other NAD+ precursors- nicotinamide riboside (NR), nicotinic acid, and nicotinamide [13]. Unlike NMN, nicotinic acid, and nicotinamide have several disadvantages in terms of their therapeutic application. Nicotinamide may cause hepatotoxicity or flushing, while a recent preclinical study suggests that it resides in the rat body for a shorter period of time compared to NMN [14,15]. Niacin or nicotinic acid is associated with adverse effects like cutaneous flushing when administered as an immediate release formulation whereas the sustained release formulations may cause hepatotoxicity [16]. Among the NAD+ precursors, NR and NMN are exceptions as fewer unfavorable side effects have been reported for these two metabolites [17]. Moreover, nicotinamide riboside is also orally bioavailable like NMN. Considering these, NMN could be proposed as a preferable therapeutic option that can be supported by several ongoing clinical trials (NCT03151239, UMIN000021309, UMIN000030609, and UMIN000025739).

Here, in this review, the biosynthetic routes and absorption of NMN are discussed followed by a comprehensive analysis of the preclinically reported pharmacological properties with their underlying mechanism of actions. This will provide an insight into the possibility of converting these successful preclinical results for the treatment of human diseases.

## 2. Biosynthesis and Mechanism of Absorption

**Figure 1:**
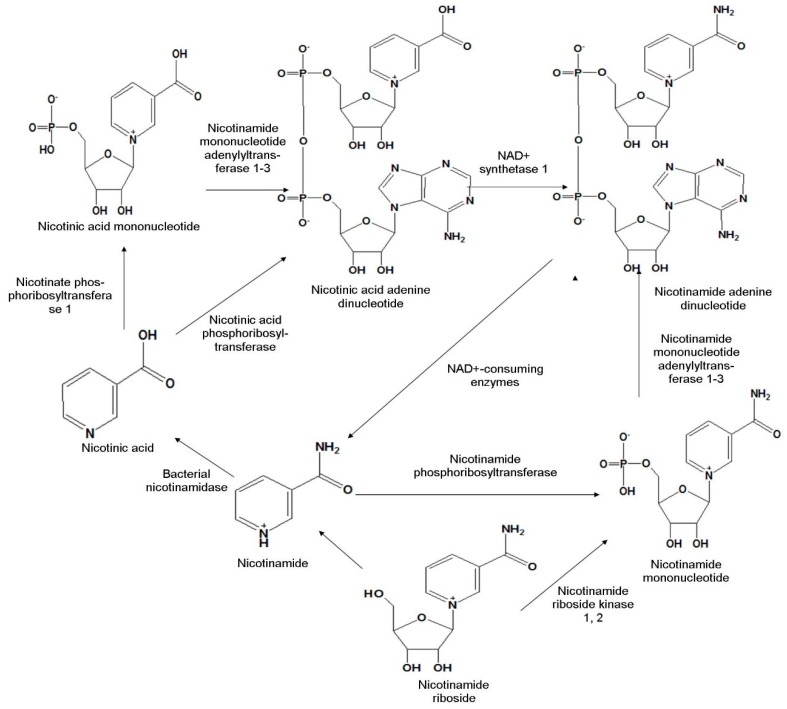
Biosynthetic pathway of nicotinamide mononucleotide in mammalian cells.

As NMN is an intermediate product of NAD+ biosynthesis, first we need to focus on the biosynthesis of NAD+ for a proper understanding of NMN synthesis. This particular biosynthetic route is important to clarify the mechanism by which the deficiency of NAD+ can be compensated. NAD+ is synthesized by three different pathways in mammalian cells-1) de novo pathway-synthesis from tryptophan, 2) salvage pathway-synthesis from either nicotinamide or nicotinic acid, or 3) conversion of NR [18]. Among these, the latter two pathways will be discussed in this review (Figure 1), as NMN is an intermediate byproduct here.

The salvage pathway is mostly predominant in mammalian cells [19,20]. In this pathway, the intermediate degradative products of NAD+ e.g., nicotinic acid and nicotinamide are reused to produce new NAD+. Most commonly, this pathway involves the conversion of the nicotinic acid to nicotinic acid mononucleotide by nicotinate phosphoribosyltransferase 1 followed by adenylation to nicotinic acid adenine dinucleotide in presence of nicotinamide mononucleotide adenylyl transferase 1, 3. Sometimes, nicotinic acid is directly converted to nicotinic acid adenine dinucleotide by nicotinic acid phosphoribosyltransferase. It is then converted to NAD+ with the help of NAD+ synthetase 1. This NAD+ is degraded to nicotinamide by NAD+ consuming enzymes, followed by conversion to NMN by the catalytic activity of nicotinamide phosphoribosyltransferase. The enzymes nicotinate phosphoribosyltransferase 1 and nicotinamide phosphoribosyltransferase both catalyze the transfer of a phosphoribosyl residue from phosphoribosylpyrophosphate (Figure 1) [21,22].

In their study with yeast and human cells, Bieganowski et al. discovered another NAD+ precursor molecule, NR, which is converted to NMN by phosphorylation with the help of nicotinamide riboside kinase (NRK1 and NRK2) [1]. The NMN formed is then enzymatically converted to NAD+ (Figure 1).

Use of prokaryotic bacteria, e.g., lysates from *Escherichia coli* (*E. coli*) with specific genotyping is shown to be a simple and cost-effective way to produce NMN [23]. In bacteria, NAD+ biosynthesis takes place in a slightly different way. Most bacteria depend on both de novo and salvage pathway (e.g., *Bacillus anthracis*) whereas some depend on either of the pathways (e.g., *Helicobacter pylori*) [24]. Some bacteria, for example, *Francisella tularensis*, a gram-negative aerobic bacterium that is the causative agent for tularemia, follows a slightly different route for NAD+ synthesis. Here, amidation of nicotinic acid mononucleotide to NMN first takes place with the help of NMN synthetase followed by adenylation to NAD+ by NMN adenyltransferase [24].

Except *Nostoc punctiforme* and *Synechocystis*, most of the cyanobacteria follow a biosynthetic pathway for NAD+ production which do not involve NMN [25]. In *N. punctiforme*, nicotinamide riboside is taken up by a PnuC-like transporter and undergoes consecutive conversion to NMN by ribosylnicotinamide kinase and then to NAD+ by nicotinamide nucleotide adenylyltransferase. On the other hand, the pathway followed by *Synechocystis* is similar to humans. Here, nicotinamide is converted to NMN and subsequently to NAD+ by the catalytic activity of nicotinamide phosphoribosyltransferase and nicotinamide mononucleotide adenylyltransferase, respectively [25].

**Figure 2:**
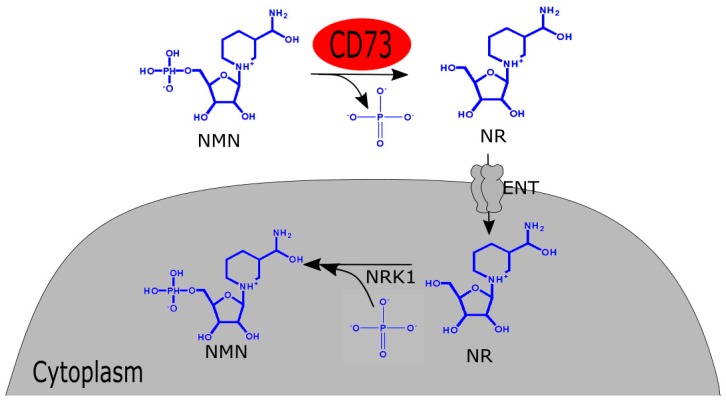
Schematic of absorption of NMN in mammalian cells. NMN: nicotinamide mononucleotide; NR: nicotinamide riboside; NRK 1: nicotinamide riboside kinase 1; ENT: Equilibrative nucleoside transporters.

The question that follows the biosynthesis is the mechanism of absorption of NMN after oral administration. After biosynthesis, NAD+ is readily absorbed through the gut wall. With the help of the murine model, it was found that the absorption of NMN from the gut into blood circulation starts within 2–3 min and within 15 min, it is completely absorbed into tissue. Then it is converted and stored immediately as NAD+ in tissues like liver, skeletal muscle, and cortex. This increase in hepatic NAD+ content persists for about 30 min [3]. After six months of NMN administration, this spiked concentration of NAD+ can be observed in the liver and brown adipose tissue, however, not in skeletal muscle and white adipose tissue [3]. Before entering the mammalian cells, NMN undergoes dephosphorylation to produce NR. Extracellular receptor CD73, with pyrophosphatase and 5’-ectonucleotidase activity, carries out the reaction. Mammalian cells have Equilibrative Nucleoside Transporters or ENT which facilitate the entry of the NR. The newly-formed NR then acts as an exogenous NAD+ precursor within the mammalian cells (Figure 2). Following this, the ubiquitously expressed NRK1 helps the subsequent conversion of NR to NMN [26].

Considering all the differences in NMN biosynthetic pathway between prokaryote and eukaryote and rapid absorption pattern of it, NMN can be conferred as one of the metabolites that have paramount significance in the turnover of NAD+.

## 3. Pharmacological Activities

NMN could open up a new horizon in modern therapeutics. This biomolecule has demonstrated numerous beneficial pharmacological activities in several preclinical disease models including myocardial and cerebral ischemia, neurodegenerative disorders like Alzheimer’s disease, and diabetes [6,7,27]. The most recent discovery of its anti-ageing, life-span prolonging property in the murine model has made NMN more attractive as a potential therapeutic candidate [28]. Most of its pharmacological actions take place by facilitating NAD+ synthesis, as direct NAD+ administration in higher dose exhibits side effects like insomnia, fatigue, and anxiety sometimes and it has poor penetration capability through plasma membrane compared to NMN [29].

### 3.1. Ischemia-Reperfusion Injury

**Figure 3:**
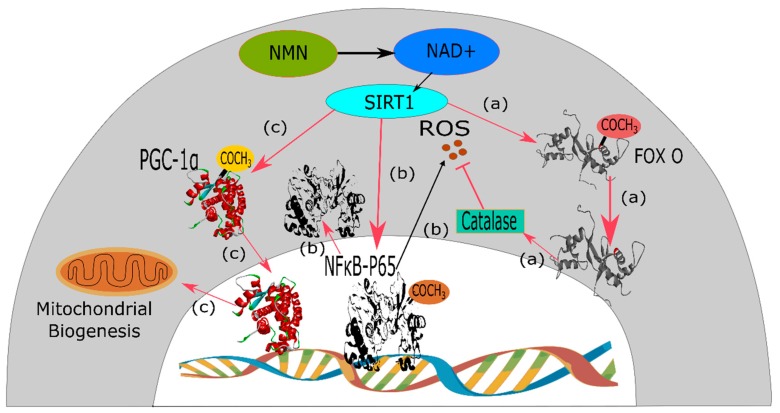
Mechanism of SIRT1 mediated pharmacological activities of NMN. NMN is converted to NAD+ intracellularly which performs physiological functions via SIRT1. (**a**) SIRT1 causes deacetylation of lysine residues of FOXO transcription factor that stimulates catalase enzyme to inhibit ROS and the chain reactions leading to ischemia-reperfusion injury. (**b**) In normal condition, p65 subunit of NFκB transcription factor complex, in its acetylated form, expresses ROS which is also responsible for insulin resistance. SIRT1, due to its inherent deacetylation activity, deacetylates p65-NFκB and thus inhibits production of ROS, which is responsible for the occurrence of type-2 diabetes mellitus. (**c**) SIRT1 also deacetylates protein PGC-1α and stimulates the expression of proteins responsible for mitochondrial biogenesis, which can be used for the treatment of Alzheimer’s disease.

Due to ischemic events, the amount of oxygen, as well as adenosine triphosphate (ATP) level in the cardiac muscle cells or cardiomyocytes, decrease. Upon further aggravation, these cardiac muscle cells undergo necrosis [30]. Reperfusion, also known as the reoxygenation process, is the event of resupplying blood to the tissue, which has previously undergone ischemia. Reperfusion causes blood to re-enter the tissue cells leading to calcium (Ca^2+^) overload by microvascular injury and production of ROS. These consecutive events cause severe tissue damage [31]. Ischemia followed by reperfusion is a deadly condition which is counteracted by a mechanism of the human body known as ischemic preconditioning or IPC [32]. IPC, an endogenous mechanism of the body, helps to revert this condition by stimulating multiple signaling mediators [33]. IPC induces activation of sirtuin1 (SIRT1) [34]. SIRT1 is a NAD-dependent class-III histone deacetylase protein which causes deacetylation of lysine residues of FoxO transcription factor that is responsible for generating oxygen free radicals. Therefore, providing a counteracting mechanism to protect the body from oxidative stress and injury due to ischemia and reperfusion. SIRT1 depends on intracellular NAD+ for its deacetylase activity [35]. Yamamoto et al. investigated the connection between nicotinamide phosphoribosyltransferase, a rate-limiting enzyme of the NAD+ salvage pathway, and IPC via activation of SIRT1 [7]. By using nicotinamide phosphoribosyltransferase +/− mice, it was found that nicotinamide phosphoribosyltransferase has a positive role of attenuation of myocardial injury following ischemia and reperfusion. Since nicotinamide phosphoribosyltransferase is the rate-limiting enzyme of NAD+ production, administration of the intraperitoneal NMN at 500 mg/kg, either 30 min before the onset of ischemia or every 6 hours during the reperfusion period for 24 hours, causes significant amelioration of ischemia-reperfusion injury by reducing the infarct size by 44% and 29%, respectively, compared to the phosphate buffer saline control. But, when this intervention was repeated against cardiac-specific KO mice, NMN intervention proved unsuccessful. Thus, it can be asserted that NMN, being the intermediate product of NAD+ biosynthesis, has the capability to activate SIRT1 (Figure 3) and thus mimics the action of IPC to ameliorate ischemia-reperfusion injury [7].

Apart from this SIRT1 mediated mechanism, another pathway shown to be responsible for this cardioprotective activity is the stimulation of glycolysis or acidosis depending on the timing of NMN delivery in respective to ischemic incidence [36]. If NMN is provided before the occurrence of an ischemic event, then glycolysis is increased that facilitates ATP production during ischemic events, thus promotes cardioprotection. In contrast, when NMN is given during reperfusion, it protects the heart by inducing acidosis, contributed by the cardiac lactate and pyruvate mainly. This causes a shutdown of mitochondrial permeability transition pore and, therefore, ensures cardioprotection [37].

NMN has also shown its therapeutic potential for the treatment of cerebral ischemia in preclinical studies. In a recent investigation by Park et al., NMN was introduced at a dose of 62.5 mg/kg in transient forebrain ischemic mice to find out the expression of different biomarkers of post-ischemic event like depletion of hippocampal NAD+ levels, accumulation of poly-ADP-ribosylation (PAR). In comparison to the effect of the control, neurologic outcome and hippocampal CA1 neuronal death after reperfusion were significantly improved by NMN treatment. Simultaneously, PAR formation and NAD+ catabolism were reduced and body temperature remained unaffected which proved that NMN treatment was solely responsible for this protective effect against ischemic brain injury [27].

Looking at the current scenario, there are only a few available intervention strategies for protecting the heart and brain from ischemia-reperfusion injury due to insufficient clinical trial data and lack of pharmacokinetic and pharmacodynamic study [38]. Again, the complex sequential events leading to this condition also limit the benefits of different surgical interventions like coronary artery bypass graft surgery and percutaneous coronary intervention [39]. NMN, being a successful entity in preclinical studies, could act as an alternative therapeutic strategy in this disorder.

### 3.2. Neurological Disorders: Alzheimer’s Disease and Intracerebral Hemorrhage

NMN has also shown promising activity in the treatment of Alzheimer’s disease as demonstrated by Long et al. It has the capability to treat underlying causes of Alzheimer’s disease, e.g., morphological abnormalities of mitochondria, decrease in oxygen consumption rates (OCR) and NAD+ content [8]. As the currently available pharmacological interventions like memantine or the cholinesterase inhibitor galantamine only aim at providing symptomatic treatment. Moreover, they cause side effects like anorexia and bradycardia, hence, NMN can play an important role since it directly targets the etiology of this disease [40]. NAD+ is recognized for its catalytic activity in aerobic respiration where oxygen is consumed by mitochondria [41]. Due to both aging and pathophysiology of Alzheimer’s disease, the availability of NAD+ decreases which leads to the decline in OCR by brain and muscle cell mitochondria [8]. Again, mitochondrial dynamics entirely depend upon fission and fusion of mitochondria. Consequently, mitochondria tend to undergo more fragmentation, i.e., increased fission and decreased fusion, leading to abnormal morphology and function of mitochondria [42,43]. In a published study by Long et al. NMN was introduced in AD chimeric APP(swe)/PS1(ΔE9) (AD-Tg) mice for mitochondrial OCR assay and on mice having fluorescent proteins tagged to neuronal mitochondria (CaMK2a-mito/eYFP) to assess mitochondrial morphology [8]. NMN was found to be effective in increasing the mitochondrial maximal OCR in transgenic AD-Tg mice compared to AD-Tg-vehicle and nontransgenic vehicle-treated group by efficiently crossing the blood-brain barrier and thus compensating the deficiency of NAD+ in mitochondria. In addition to increasing maximal OCR, NMN was successful to increase OCR significantly following the addition of ADP (for the initiation of state 3 respiration) in AD-Tg NMN treated mice compared to non-transgenic one, although the basal OCRs were similar. NMN also activated SIRT1 via NAD+ biosynthesis by salvage pathway, which, in turn, stimulated the deacetylation of target protein PGC-1α (Figure 3), responsible for mitochondrial biogenesis [44]. Again in CaMK2a-mito/eYFP mice, the NMN treated group showed reduced fragmentation and increased length of mitochondria, evidenced by an increase in rod shape and tubular length, which ensured the integrity of mitochondria [8].

The amyloid beta oligomer, also known as Aβ or A-beta, is a type of neurotoxic protein which forms amyloid plaques in the brains of Alzheimer patients [45]. It is also responsible for inducing depression of hippocampal long-term potentiation or LTP [46]. NMN successfully inhibited Aβ oligomers induced LTP by 140% compared to that of baseline. On organotypic hippocampal slice cultures (OHCs), NMN decreased Aβ oligomers induced cell death by 65% in Aβ oligomer infusion AD model rats (dose 500 mg/kg, intraperitoneally) indicating the improvement in cognitive function [45]. This finding confirms the previous studies where NMN has been proved for its potential therapeutic application in Alzheimer’s disease. With further studies regarding the dosage regimen and subsequent clinical trials, it could be a promising therapeutic intervention for Alzheimer’s disease in human.

Intracerebral hemorrhage (ICH) is another neurological disorder, responsible for about 10–15% of all strokes, where NMN can improve the disease condition [47]. The injury caused by ICH occurs in two stages: at first, mechanical damage to neighboring tissues takes place by hematoma formation that is followed by secondary damage via pathological alterations (e.g., cytotoxicity, excitotoxicity, activation of inflammatory pathways causing neuroinflammation) caused by hematoma [48]. A study on collagenase-induced ICH mouse model, upon exposure to 300 mg/kg intraperitoneal dose of NMN after 30 min of ICH episode, showed that NMN treatment increased intracerebral NAD+ concentration at two and six-hour post-ICH and provided protection against amyotrophic lateral sclerosis and ischemic stroke. Furthermore, NMN treatment resulted into significant improvement, measured by a decrease in edema, neuronal death, ROS content, neurological inflammation, expression of intercellular adhesion molecule-1, neutrophil infiltration and microglia activation in the affected area of the brain. Although this intervention failed to reduce hematoma volume and hemoglobin content [49], it successfully improved the conditions mediated by ICH which suggests that NMN can be used for the treatment of ICH.

### 3.3. Diabetes

NMN has also shown potential to be used as a therapeutic for the treatment of diabetes. Insulin resistance is the characteristic feature of type 2 diabetes which occurs due to oxidative stress, augmented inflammatory response, impaired lipid metabolism- all of which can be ameliorated by NAD+ [6]. High-fat diet and ageing contribute to the predisposition of this particular type of diabetes, one of the common mechanisms being the reduction of NAD+ [6]. High-fat diet, consisting mainly of saturated fats, can decrease nicotinamide phosphoribosyltransferase level and ultimately reduce NAD+ in the liver and white adipose tissue [6]. On the other hand, ageing influences the decrease of NAD+ level in the pancreas, white adipose tissue, skeletal muscle, and liver to a greater extent compared to the younger population [6]. This NAD+ acts as a safeguard against various physiological disorders. Glutathione S-transferase, a protective agent against oxidative stress from lipid peroxidation products, is restored by NAD+ [50]. Furthermore, expression of interleukin 1β (IL-1β) and S100 calcium-binding protein A8 and A9, the two targets of immune and inflammatory mediator nuclear factor kappa B (NF-kB), are downregulated by NAD+ [51]. This NF-kB is also responsible for causing insulin resistance. Activation of SIRT1 by NAD+ promotes deacetylation of p65 component of NF-kB (Figure 3) and thus prevents insulin resistance [52]. To revert the high-fat diet and ageing induced type 2 diabetes, Yoshino and his associates administered NMN, at 500 mg/kg/day intraperitoneally for 7 and 10 consecutive days in high-fat diet fed female and male diabetic mice, respectively. They found a significant improvement in insulin intolerance in both female and male mice, while female mice showed improvement to a greater extent [6]. In the case of age-induced diabetes, the same single dose and 11 consecutive doses of intraperitoneal NMN ameliorated glucose intolerance in diabetic male and female mice respectively. NMN did not hamper glucose homeostasis in non-diabetic normal mice whereas, energy utilization solely from glucose and improvement of hyperlipidemia in aged diabetic mice were ensured by this intervention. It also improved inflammation mediated islet cell dysfunction by increasing extracellular nicotinamide phosphoribosyltransferase concentration through suppression of IL-1β from islets cells and thereby restored insulin secretion from beta cells [53].

### 3.4. Obesity and Its Related Complications

NMN can reduce age-associated weight gain in a dose-dependent manner, supported by a research on mice [3]. In this study, 100 and 300 mg/kg dose of NMN, applied throughout a duration of 12 months, was capable of reducing body weight by 4% and 9%, respectively, compared to the control group without any compromise to growth and appetite. There is an interconnection between the pathology of obesity and diabetes. Obesity exerts negative health effects through alteration in biochemical pathways that cause mitochondrial dysfunction. The decreased ATP production through changes in NAD+ and NADH level by dysfunctional muscle and hepatic mitochondria leads to insulin resistance and type-2 diabetes [54,55,56]. NAD+ helps to replenish the cellular energy level by stimulating mitochondria to generate ATP [57]. As mentioned before, SIRT1 utilizes NAD+ as a cofactor to improve mitochondrial biogenesis, (Figure 3) which is hampered due to obesity [58]. In a recent study, NMN was administered in high-fat diet (HFD) induced obese mice to assess and compare the capability of improving the NAD+ content with that of treadmill exercise, at an average of 15 m/min taken for 45 min and continued six days per week for six weeks. NMN treatment at a dose of 500 mg/kg body weight daily for 17 days was successful in increasing the NAD+ content in both muscle and liver, which was previously reduced by HFD-induced obesity, whereas exercise increased NAD+ in muscle only [59]. NMN treatment also improved metabolic disorders like glucose intolerance and reduced hepatic citrate synthase activity, similar to that of exercise [59]. In light of the above discussion, it can be inferred that NMN can improve certain metabolic complications associated with obesity, although this effect on many other obesity-related metabolomic complications like the non-alcoholic fatty liver disease is yet to be explored [59].

### 3.5. Ageing

Geroscience is the field that deals with the relationship between ageing and the diseases accompanying these [60]. The process of ageing comes along with some age-related complications. Ageing is a natural human phenomenon characterized by downregulated energy production by mitochondria, as previously mentioned, owing to the depletion of NAD+ in multiple organs like pancreas, skeletal muscle, liver, skin, adipose tissue and brain [3,61,62]. Aside from the decrease in the mitochondrial function, ageing is also associated with other biological alterations like-DNA damage, cognitive impairment, sirtuin gene inactivation which can be retreated by NAD+ [63]. The age-related depletion of the NAD+ count, specifically that of nuclear origin, is also accounted for disruption of mitochondrial regulation of PGC-1α/β-independent pathway of oxidative-phosphorylation, leading to pseudohypoxia. This can be reversed by increasing the amount of NAD+ [64]. NAD+ has been reported for its ability to regenerate cells like muscle stem cells in older mice. Another precursor of NAD+, NR, has also been attributed for its ability to induce neurogenesis, halt the decrease in melanocyte stem cells and increase the lifespan in mice slightly [65]. Being the precursor of NAD+, NMN can provide these beneficial effects as well.

Decreasing NAD+ level is also associated with age-related DNA damage. NAD+ binds to the nudix homology domain of various proteins. Although the specific function of nudix homology domain is yet to be found, studies have shown that the Deleted in Breast Cancer protein1 (DBC1) possesses this domain, which inhibits the DNA repairing protein PARP1 [66]. NAD+ binds to nudix domain of DBC1, thus the inhibition on PARP1 is reversed. With ageing, NAD+ level declines, so PARP1 loses its capability to repair DNA. This phenomenon was assessed on old (30-month-old) and young mice (6-month-old) with gamma radiation-induced DNA damage. The mice were treated with an intraperitoneal NMN at 500 mg/kg/day for one week. After the experimental period, it was found that NMN increased hepatic NAD+ concentration as well as PARP1 activity in repairing DNA damage [67].

With the course of ageing, the pathophysiological changes in different cells and tissues like- eye and bone have become apparent. The lacrimal gland becomes less capable of producing tears, whereas, in some cases, light-colored spots in the fundus of the eye due to an age-dependent pileup of subretinal microglia and macrophages takes place [67,68].NMN, at a dose of 100 and 300 mg/kg/day, was found to be effective in reducing these spots in the fundus of the transgenic mouse strain C57BL/6N which contains the rd8 mutation, responsible for these abnormalities [3]. A 12-month NMN administration also increased tear production in a dose-dependent manner, in which the 300mg/kg/day dose was capable to increase the level of tear production comparable to that of maximal tear production of mice lifetime [3]. Thus, it substantiates its capability of reverting the optical abnormalities due to ageing. In addition, bone density depletion, a characteristic attribute of age-associated physiological change, was reverted significantly by NMN in a dose-dependent manner [3].

Senescence of the vascular system is another common occurrence during the process of aging. It is accompanied by oxidative stress imposed by the free radicals. NMN was found to be effective in reversing these conditions when tested on mice model. De Picciotto and his co-researchers conducted a study on this particular field to analyze the efficacy of NMN [69]. Vascular functionality was assessed in terms of carotid artery endothelium-dependent dilation (EDD); while nitric oxide-mediated EDD was used to analyze vascular oxidative stress. The older control mice (26–28 months) showed diminished functions on both of these parameters compared to the young control (4–8 months). Following an 8-week oral NMN intervention at 300 mg/kg/day on male C57B1/6 mice, both carotid artery EDD and nitric oxide EDD were restored to normal level, like that of young control mice. Again, the conditions of older mice like the abundance of oxidative stress marker nitrotyrosine, reduced elastin and vascular SIRT1 activity, all were reversed by NMN administration. Moreover, when arteries were incubated with NMN for 48 hours, oxidative stress was reduced due to a 50% increase in manganese superoxide dismutase and a threefold increase in NAD+ levels [69].

The expressions of certain genes in metabolic organs like skeletal muscle, white adipose tissue, liver and immunological functions also start to decline with ageing. Mills et al., while conducting microarray assessment, found that 76.3% of 300 compromised genes of skeletal muscle, 73.1% of 360 compromised genes of white adipose tissue, and 41.7% of 513 compromised ones from liver were upregulated by NMN treatment on mice [3]. Not only this, the increased expression of immune cells from the immunometabolic system, specifically that of white adipose tissue, improvement in hematological conditions like a decrease in neutrophils and increase in lymphocyte, cytokine activity, leukocyte activation, are the outcomes of NMN treatment upon aged mice [3,70]. As stated earlier, increase in body weight and obesity-related complications like a decrease in energy metabolism and locomotor activity, age-dependent insulin insensitivity and higher triglyceride levels are also associated with ageing. These conditions were reversed by 12-month NMN intervention [3].

The success of NMN in preclinical study instigated the researchers of Keio University School of Medicine in Tokyo and Washington University School of Medicine of St. Louis to initiate a collaborative research program for the phase I NMN clinical trial [28]. The goal of this study was to assess the safety and to find out the bioavailability of NMN in the clinical model. A positive outcome from this study could curve a new direction for the long-awaited treatment strategy of ageing.

## 4. Nicotinamide Mononucleotide or Nicotinamide Riboside: Which One is Better

Among the diverse type of NAD+ precursors, until now only NMN and NR presented better pharmacokinetic and pharmacological property. Accordingly, these two intermediates are now widely utilized for clinical trials. Still, the question remains: Which one is better? Researchers in favor of both the intermediates have their supporting arguments. NR is highly available in normal human diet and its cellular permeation is simple as NR does not require any conversion to other intermediates. There are safety studies for NR administration while NMN is yet to prove the safety of human consumption. In a 12-week study of 2000 mg/day NR supplementation to assess the efficacy of improvement of glucose metabolism and insulin sensitivity in obese patients, although NR did not improve glucose metabolism and insulin sensitivity, NR treatment was safe [71]. On the other hand, NMN has quite a few strong advantages of its own. While assessing the efficiency to treat Friedreich’s Ataxia (FRDA), a rare inherited childhood heart disease, NMN was found successful where NR treatment failed [72,73]. In this disease, increased acetylation of a mitochondrial protein namely frataxin and impaired SIRT3 activity results in cardiac hypertrophy. An intervention of 500 mg/kg NMN 2 times per week for six weeks on FXN-KO mice caused improvement in diastolic function and normalized systolic function compared to that of normal saline-treated control mice. This positive effect is mediated by increasing the deacetylase activity of SIRT3 on frataxin [72], while in another study, NR at a dose of 10 mg/kg for five weeks on FXN KO mice neither improved SIRT3 activity nor cardiac function [73].

In case of treatment of cognitive impairment in Alzheimer’s disease, for which β amyloid plaque is thought of as the main culprit, NMN was potent enough to reduce the burden of amyloid β plaque by reducing its production. During a 6-month long NR intervention at a dose of 12 mM in drinking water on 3xTgAD/Polβ+/− mice, it was observed that DNA damage, neuroinflammation, and apoptosis of hippocampal neurons were significantly decreased whereas SIRT3 activity in brain was increased leading to improvement of cognitive function, although, there was no impact on amyloid β accumulation [74]. In contrast to this, subcutaneous administration of 100 mg/kg NMN for 28 days reduced the loss of synapses, inflammation and improved neurobehavioral activities by reducing amyloid β build up in the brain. The mechanism behind this was the inhibition of amyloidogenic amyloid precursor protein (APP) and stimulation of nonamyloidogenic APP by NMN [75].

It is very hard to delineate the borderline between the efficiency of NMN and NR. It is reasonable to conclude that both of them share some overlapping activities, as well as their own positive and negative impacts.

## 5. Future Prospects

As a precursor molecule of NAD+ in our biological system, NMN can play an important role in the treatment of various disease states. NAD+ was found to stimulate cell survival in response to genotoxic stress like exposure to benz(a)anthracene, and benz(a)pyrene [76]. The reason behind cell death due to genotoxicity is the hyperactivation of NAD+ dependent mitochondrial DNA repair enzyme- PARP-1 which utilizes and thus decreases NAD+. It also causes translocation of apoptosis-inducing factor (AIF) from mitochondrial membrane to the nucleus [77,78,79]. By providing NAD+, cell survival can be significantly improved. Whether NMN can serve as a substitute for NAD+ to improve cell survival could be an important field of research.

This particular mechanism is also responsible for beta cell destruction leading to type 1 diabetes [80]. When beta cells of pancreatic islets are exposed to beta cell toxin, namely streptozocin or oxidative stress, PARP-1 utilizes the cellular NAD+ storage to revert DNA strand break. Consequently, ATP production and protein synthesis are reduced which triggers beta cell death [81,82]. NMN could be used to compensate the ATP shortage, thus enabling the beta cells to survive.

Aside from the two aforementioned fields of research, the association of NAD+ with enzymes like mono-ADP-ribosyltransferases and sirtuin enzymes, which control apoptosis, DNA repair, stress resistance, metabolism, and endocrine signaling, could also be an area of interest [13]. As NAD+ metabolism can be a potential target for abnormalities related to these biological processes, NMN can be of therapeutic benefit.

## 6. Conclusions

Although NMN has shown significant beneficial pharmacological activities in preclinical studies which the scientists have been searching for a long time, it still lacks sufficient clinical and toxicological data. The high manufacturing cost of NMN causes an increase in the ultimate price that creates a burden from the patients’ perspective. Despite having these drawbacks, NMN could still be a potential chemical entity, to be used as a therapeutic agent in Alzheimer’s, diabetes, cardiovascular diseases. Some capsule formulations of NMN is already available in the market. With advanced clinical studies along with the exploration of newer pharmacological applications, NMN could be an ‘all-in-one’ intervention strategy, transpiring a new era of therapeutic approach in medical science.
